# Patient Engagement Initiatives in Clinical Trials: Recent Trends and Implications

**DOI:** 10.1007/s43441-021-00306-8

**Published:** 2021-06-07

**Authors:** Shalome Sine, Annick de Bruin, Kenneth Getz

**Affiliations:** 1 Center for Information and Study on Clinical Research Participation, One Liberty Square, Suite 1100, Boston, MA 02109 USA; 2 Tufts University School of Medicine, Boston, MA USA

**Keywords:** Clinical trials, Patient engagement, Recruitment, Enrollment, Retention, Participation burden, Technology

## Abstract

**Background:**

As clinical trial protocol designs become more complex and eligible patient populations narrow, it is becoming increasingly difficult to recruit participants and retain them for the duration of the trial. This study surveyed clinical trial participants to learn about the prevalence and impact of new technologies and other supportive solutions designed to improve patient engagement and retention. Patient perceptions of these convenience-enhancing solutions and how they have changed since our last study in 2017 were examined.

**Methods:**

Based on 12,451 responses to a global online survey collected in 2019, we conducted an analysis of respondents who used convenience-enhancing solutions during their participation in a clinical trial.

**Results:**

We found that the prevalence of convenience-enhancing solutions is increasing and that their use correlates with high ratings for clinical trial satisfaction, as well as with high ratings for care and attention received during the trial.

**Conclusions:**

A wide range of strategies and tactics are needed to reduce barriers to participation and improve retention. The use of convenience-enhancing solutions can help reduce these barriers. The solutions are also particularly popular among under-represented populations, revealing further potential opportunities to increase patient engagement specifically among these groups.

## Background

Recruitment and retention have long been key challenges in clinical trials and over the past several years, clinical research sponsors have begun introducing new technologies and other supportive solutions designed to boost enrollment, patient engagement, and overall satisfaction, care, and attention ratings [1]. Some of these efforts have been directed toward encouraging physician participation in clinical research [2]; however, most have focused on reducing patient participation barriers [3]. Generally, these new technologies and solutions aim to increase patient convenience by mitigating logistical burdens and range from the relatively low-tech, such as concierge services, childcare, and pre-paid travel and accommodations, to the considerably more high-tech, such as smartphone apps and wearables [4, 5]. Recently, the pandemic has fueled even greater interest in technology to facilitate remote monitoring and support of study participants [6, 7].

This study examined the attitudes, perceptions, and experiences of clinical trial participants to learn about the prevalence and impact of certain patient engagement strategies and how opinions regarding these convenience-enhancing solutions have changed since our last study in 2017.

## Methods

The Perceptions & Insights study is a longitudinal study designed to investigate trends and overall public opinion regarding clinical research. Since 2013, we have administered global, online, surveys once every 2 years.

Each biennial survey is written and developed by a work group consisting of representatives from patient advocacy groups, pharmaceutical companies, investigative research clinics, and contract research organizations. After ethical review through the New England IRB, the survey is translated into several languages to reach its global target population.

In 2019, organizations including Acurian, Clariness, Continuum Clinical, CureClick, and IQVIA collaborated with CISCRP to engage respondents using a convenience sampling strategy. These organizations reached out to their networks of patients and the general population. Outreach efforts resulted in a total of 12,451 respondents completing the survey. A similar sampling and respondent engagement strategy was used in 2017. While outreach and dissemination efforts in 2017 and 2019 were distinct, the study was conducted in a similar manner across both survey years.

Of those who completed the survey in 2019, 55% (6846) resided in North America, 6% (761) in South America, 27% (3419) in Europe, 1% (103) in Africa, and 11% (1322) in the Asia/Pacific region (see Table 1). Of the total sample size, 29% (3,654) had clinical research participation experience for a range of medical conditions. Top mentions for conditions include 11% (414) participating in a clinical trial for diabetes, 7% (264) for heart/cardiovascular conditions, 5% (176) for arthritis, 5% (168) for mental health conditions, 5% (167) for blood disorders, and 4% (141) for oncology. Of those who participated, 17% (626) were healthy volunteers.

**Table 1:** Respondent profile

Variable	2017^a^, % (*n* = 12,427)	2019^b^, % (*n* = 12,451)
Gender		
Female	59	55
Male	40	44
Region		
North America	46	55
South America	7	6
Europe	28	27
Asia–Pacific	14	11
Africa	5	1
Age (years)		
18–34	13	13
35–44	11	14
45–54	19	20
55–64	27	26
≥ 65	29	26
Race (top mentions)		
White	81	78
Black	6	6
Asian	5	11
Ethnicity (top mentions)		
Non-Hispanic/Latino	88	85
Hispanic/Latino	8	14
Education		
No school/primary education only	2	2
Some/completed high school	24	32
Some/completed college	58	50
Some/completed postgraduate	16	17

^a^2017 CISCRP Perceptions & Insights Study; some columns may not total 100 because “other” responses are not shown

^b^2019 CISCRP Perceptions & Insights Study; some columns may not total 100 because “other” responses are not shown

For each of these subgroup sample sizes, the margin of error ranges from ± 0.2 to 1.22% at a confidence level of 95%. Potential biases related to sampling and methods used can be found in the Limitations section.

All survey respondents were asked about their perceptions and opinions regarding clinical research. Those who had participated in clinical research were asked further questions about their experiences. Clinical research participants were asked which, if any, of 11 convenience-enhancing solutions were offered during their trial. These solutions consisted of electronic consent form signing, text messaging from the study center, smartphone apps, wearable devices, social media, concierge services, home/office study visits, study visits with the patient’s regular doctor, childcare, video conferencing with the study doctor, and satisfaction surveys. The first nine of these solutions were included in the same survey question in 2017, allowing for longitudinal comparison of their prevalence. Video conferencing with the study doctor and satisfaction surveys were added to the list of convenience-enhancing solutions for the 2019 study.

Solution usage levels were investigated across subgroups defined by age, region, race and ethnicity, study phase, study type, and internet/social media use habits. Statistically significant results were identified at a 95% confidence interval and null hypotheses were rejected at *P*-values of less than 0.05.

Questions related to study experience satisfaction were analyzed to test the hypothesis that the use of these solutions positively impacts clinical trial experiences. Results were analyzed as a function of solution prevalence, as well as longitudinally. Convenience-enhancing solutions which demonstrated associations with significant increases in study satisfaction outcomes were included in this report.

Future surveys will continue to include questions on the prevalence of these solutions so that longitudinal analyses can create a broader view of changes in their usage over time.

## Ethical Review

The 2019 study was reviewed and deemed exempt by New England IRB, a Division of Western Institutional Review Board.

## Results

### Travel is Identified as Top Study Participation Burden

When prompted on the most burdensome aspects of their participation, survey respondents ranked traveling to the study clinic as the top burden, with 3 out of 10 (29%) indicating that it was “somewhat” or “very burdensome.” The next most burdensome aspects were the length of the study visits and undergoing diagnostic tests (e.g., X-rays, MRIs), each with 21% indicating this was “somewhat” or “very burdensome.”

### Important Supportive Services Can Help Mitigate These Participation Burdens

When considering participation in clinical research, several solutions and services were identified as most important to reduce these burdens. The top responses indicated that patients want supporting information on managing their health condition in general (with 50% indicating that this was “very important”), supporting information on the clinical research study (e.g., study guides, pamphlets) (48%), the opportunity to complete a satisfaction survey on the clinical research study experience (41%), the availability of concierge services (34%) such as transportation to and from the study clinic, and mobile applications (e.g., electronic surveys, visit reminders sent via text) (31%).

### Convenience-Enhancing Solutions Usage Grows

The use of convenience-enhancing solutions has grown substantially over the past 2 years. Technology-based options were the fastest growing category, with the use of smartphone apps and wearable devices in clinical trials up 5% and text messaging up 3% since 2017 (see Table 2). Still, only 21% of survey respondents who had participated in a clinical trial reported the use of text messaging, and even fewer used smartphone apps (15%) or wearables (13%). Concierge services, which might include transportation to and from study clinics, exhibited strong growth as well, while the use of other supportive services, such as the provision of childcare, increased more modestly. Notably, the proportion of those who indicated that none of the given solutions were used during their participation fell 13%, from 40% in 2017 to 27% in 2019.

**Table 2:** Changes in convenience-enhancing solution use, 2017 to 2019

Variable	2017^a^ (*n* = 2194)	2019^b^ (*n* = 3654)
Informed consent on an electronic tablet (et., iPad)*	17	15
Text messaging (for study visit reminders or study instructions)*	18	21
Smartphone apps for study data collection**	10	15
Wearable devices**	8	13
Social media	4	5
Concierge services**	7	11
Some or all of my study visits were conducted at my home or my office	7	8
Some or all of my study visits were conducted at my regular doctor’s office rather than the study doctor’s office	12	12
Childcare or childcare reimbursement*	1	2
Surveys to collect information on my clinical trial experience	–	29
Video conference with the study doctor	–	4
None of the above**	40	27

Values are a percentage of the total

*P < 0.05, **P < 0.001

^a^2017 CISCRP Perceptions & Insights Study; multiple responses allowed; excludes ‘Other’

^b^2019 CISCRP Perceptions & Insights Study; multiple responses allowed; excludes ‘Other’

### Use of Convenience-Enhancing Solutions Varies by Age and Social Media Habits

Younger survey respondents were more likely to use convenience-enhancing solutions as part of a clinical trial compared to older age groups. The difference was most prominent in the use of technology-based solutions, such as social media, which showed a 16% gap in usage between the youngest and oldest age groups (at 17% and 1% usage, respectively; *P* < 0.005). Similar usage gaps were detected for smartphone apps, video conferencing, and wearable devices. These results mirror our findings in 2017, when 30% of those 18 to 34 used smartphone apps, compared to just 4% of those 65 and older.

Younger participants were also the biggest users of non-technology-based solutions. Concierge services, study visits conducted by the participant’s regular doctor, and study visits conducted in the participant’s home or office were all used more by those in the youngest age category, with regular doctor study visits demonstrating a 15% higher use among those 34 years of age and under compared to those 65 years of age and older (*P* < 0.005). Furthermore, only 9% of those in the youngest age group reported using none of the listed solutions, compared to about one-third of the oldest age group.

As might be expected, those who used social media “sometimes” or “always” in their daily lives, outside of a clinical trial environment, were more likely to use technology-based solutions, including eConsent, text messaging and social media when participating in a clinical trial. For example, 17% of those who used social media “sometimes” or “always” in their daily lives reported that they had an electronic consent form as part of their clinical trial, significantly more than just 11% of those who used social media “never” or “rarely” (*P* < 0.01).

### Region, Race and Ethnicity Also Impact Usage of Convenience-Enhancing Solutions

The use of convenience-enhancing solutions was most prevalent in South America and in the Asia/Pacific region, with these areas showing elevated use of study visits conducted at a participants’ home, office, or regular doctor’s office, as well as technology-based options, such as wearable devices and social media. For example, 22% of those in South America and 22% of those in the Asia/Pacific region used wearable devices during their clinical trial, with both regions showing a significantly higher use of wearable devices compared to just 10% of those in North America (*P* < 0.001). Only 10% of South American participants and 14% of participants from the Asia/Pacific region indicated that they used no convenience-enhancing solutions, while this number jumped to 29% and 28% for North America and Europe, respectively.

Our 2019 study results also indicated significant growth in the use of some convenience-enhancing solutions in the Asia/Pacific region over the past 2 years. For example, smartphone app use grew from just 4% in the Asia/Pacific region in 2017 to 26% in 2019, the highest of any region.

In 2019, we also found variations in solution usage based on ethnicity. Results indicate that Hispanic respondents are consistently more likely to use convenience-enhancing solutions compared to non-Hispanic survey respondents. For instance, 18% of Hispanic survey respondents used concierge services compared to only 10% of non-Hispanic survey respondents (*P* < 0.001). These results are consistent with those from 2 years ago; in 2017, 30% of those who were Hispanic used text messaging compared to just 18% of those who were non-Hispanic (*P* < 0.01).

Differences across various race subgroups were also identified. White survey respondents were consistently less likely to report the use of convenience-enhancing solutions compared to Black or Asian survey respondents. For example, 26% of those who were Asian and 23% of those who were Black used smartphone apps during participation, with both race subgroups showing significantly higher use of smartphone apps compared to just 13% of those who were White (*P* < 0.001). We found similar results in the 2017 study, when solution use was higher among Black survey respondents in most categories compared to those who were White. For example, in 2017, 15% of those who were Black used wearable devices, compared to just 7% of those who were White (*P* < 0.01).

### Satisfaction, Care and Attention Ratings Highest Among Those in Trials with Convenience-Enhancing Solutions

**Fig. 1:**
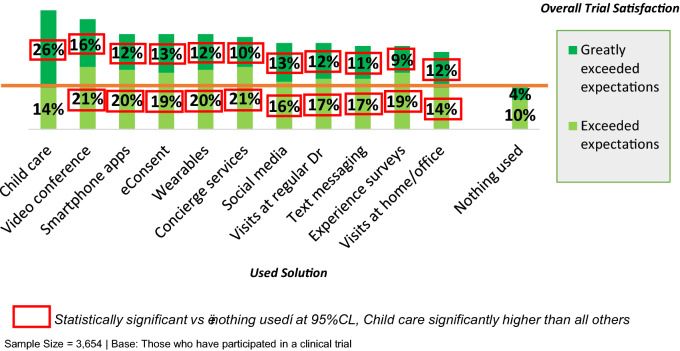
Impact of convenience-enhancing solutions on ratings of satisfaction

Satisfaction ratings were highest among those who used convenience-enhancing solutions in their clinical trial (see Fig. 1). For example, 40% of those whose clinical trial offered childcare indicated that the study “exceeded” or “greatly exceeded” their expectations, compared to just 14% of those who did not use any solutions during participation (*P* < 0.001). A similar effect could be seen across all of the convenience-enhancing solutions, as the proportion who mentioned that the study “greatly exceeded” their expectations was consistently significantly higher among those who used any solution compared to those who did not.

Care and attention ratings were also higher among those who used convenience-enhancing solutions compared to those who did not. Those who used technology such as smartphone apps and video conferencing, during their clinical trial participation were most likely to say that the care and attention they received during their participation was better than the standard of care. Notably, those who indicated that they were offered study visits at their home or office, or through at their regular doctor’s office, were also more likely to say that they received better care and attention compared to those who said that no solutions were used. These impacts are consistent over time, and similar associations could be found in 2017, when care and attention ratings were highest among those who used electronic informed consent forms and wearable devices.

## Discussion

As clinical trial protocol designs increase in complexity, it is becoming more difficult to recruit eligible patient participants and retain them for the full study durations [8–11]. In response to these challenges, clinical research and clinical care professionals have begun introducing a variety of technologies and other services aimed at improving patient engagement [9, 10].

The results of our 2019 Perceptions & Insights study illustrate that the prevalence of convenience-enhancing solutions is increasing and that their use correlates with high ratings for clinical trial satisfaction, as well as with high ratings for care and attention. Technology-based solutions, such as smartphone apps and wearable devices, were particularly well received. These results suggest that it would be beneficial for researchers to include a variety of convenience-enhancing solutions in their clinical trial designs. As such, patient participants can choose the options that are most helpful to them, based on lifestyle factors such as work schedules, social media habits, childcare needs, and caregiver involvement [10].

We also found evidence that new technologies may be more popular among those who are Black and those who are Hispanic. These insights reveal potential opportunities for improving patient engagement among under-represented populations. Focusing efforts this way is critical for enhancing diversity in clinical trials. That, in turn, will lead to a better understanding of variability in drug response and help ensure patients get the treatments that best meet their needs. Indeed, as part of the 2012 Food and Drug Administration Safety and Innovation Act (FDASIA 907), the FDA’s Center for Drug Evaluation and Research (CDER) has begun publishing information on the diversity of participants in clinical trials [12]. In 2018, an analysis of this data found that Black and Native American populations are under-represented in clinical trials of new drugs, even when the treatment is aimed at a type of cancer that disproportionately affects them [13]. Though addressing these disparities will require wide-reaching industry effort, our results indicate that underrepresented populations consider participation barriers more burdensome and may especially benefit from convenience-enhancing solutions. Providing these options may be an essential component of successful inclusion strategies.

Even though clinical trial participants are using these solutions more than ever before and satisfaction ratings are highest among those who do, the overall prevalence of these technologies and services remains low. The cost and the highly regulated healthcare environment are likely to remain as significant challenges; however, the growing acceptance of patient-centric study designs will help bridge this gap.

Furthermore, the COVID-19 pandemic has significantly transformed the way that clinical trials operate [14]. As sites react to disruptions caused by the pandemic and identify ways to comply with physical distancing regulations, we expect to see an increase in the adoption of convenience-enhancing solutions, especially those which are technology-based such as virtual study visits [14]. We plan to investigate the effects of the pandemic on the use of convenience-enhancing solutions and any associated impacts to clinical trial participation experiences in an upcoming global study.

It is important to additionally consider that improved patient recruitment and retention enhance the quality and efficiency of clinical trials and may help accelerate the drug development process [15]. Our results suggest that a wide range of strategies and tactics, from concierge and childcare services to surveys collecting feedback about the clinical trial experience, reduce barriers to participation, improve patient engagement, and ultimately, can help bring new treatments to market faster.

## Conclusions

Continuous assessment of public and patient attitudes, perceptions, and experience in clinical trials leads to insights that can help researchers design and implement effective engagement strategies and tactics. After all, in order to run efficient, patient-centered clinical trials, it is vital to reduce participation burdens wherever possible. Convenience-enhancing solutions, whether technology-based or otherwise, have the potential to reduce some of the barriers to and burdens of participation. Our findings also show that convenience-enhancing solutions positively impact participant experience ratings. Leveraging these insights may allow the industry to address recruitment and retention barriers while improving the participation experiences of patients.

## Limitations

The results of the 2019 CISCRP survey, and those of past surveys, are all based on responses from convenience samples. Although the number of international responses is large, the surveys were all conducted online among adults who self-identify as people seeking health-related information and who have opted in to receive email communications and invitations. As such, the results of these surveys should be viewed with some caution as they reflect sampling bias and may not be representative of the views of the entire global population, most notably, those who cannot access, receive, and read online solicitations and communications. Finally, a large proportion (55%) of respondents in 2019 resided in North America as compared to 46% of respondents in 2017 which may influence the overall conclusions that we have drawn.

## References

[1] Khozin S, Coravos A (2019). Decentralized trials in the age of real-world evidence and inclusivity in clinical investigations. Clin Pharmacol Ther.

[2] Rahman S, Majumder A, Shaban S, Rahman N, Ahmed SM, Abdulrahman K (2011). Physician participation in clinical research and trials: issues and approaches. Adv Med Educ Pract.

[3] National Academies of Sciences, Engineering and Medicine, Division of Health and Medicine, Policy Board on Health Sciences, Forum on Drug Discovery, Development and Translation, Shore C, Khandekar E, et al. Opportunities to improve clinical trials. National Academies Press (US); 2019. www.ncbi.nlm.nih.gov (cited 21 Oct 2020). https://www.ncbi.nlm.nih.gov/sites/books/NBK548971/. Accessed 7 Dec 2020.

[4] CenterWatch Staff. Sanofi launches digital clinical trials to improve recruitment and reduce trial times. Centerwatch.com. CenterWatch; 2017 (cited 21 Oct 2020). https://www.centerwatch.com/articles/13976. Accessed 7 Dec 2020.

[5] PAREXEL. Using patient sensors to bring the future of clinical trials to patients, today. www.clinicalleader.com. PAREXEL; 2020 (cited 21 Oct 2020). https://www.clinicalleader.com/doc/using-patient-sensors-to-bring-the-future-of-clinical-trials-to-patients-today-0001. Accessed 7 Dec 2020.

[6] Tan AC, Ashley DM, Khasraw M (2020). Adapting to a pandemic—conducting oncology trials during the SARS-CoV-2 pandemic. Clin Cancer Res.

[7] Izmailova ES, Ellis R, Benko C (2020). Remote monitoring in clinical trials during the COVID-19 pandemic. Clin Transl Sci.

[8] Getz KA, Campo RA (2017). Trends in clinical trial design complexity. Nat Rev Drug Discov.

[9] Marquis-Gravel G, Roe MT, Turakhia MP, Boden W, Temple R, Sharma A, et al. Technology-enabled clinical trials: transforming medical evidence generation. Circulation. 2019;140(17):1426–36 (cited 19 April 2020). https://www.ncbi.nlm.nih.gov/pubmed/31634011. Accessed 7 Dec 2020. 10.1161/CIRCULATIONAHA.119.040798 31634011

[10] Sommer C, Zuccolin D, Arnera V, Schmitz N, Adolfsson P, Colombo N (2018). Building clinical trials around patients: evaluation and comparison of decentralized and conventional site models in patients with low back pain. Contemp Clin Trials Commun.

[11] Tufts Center for the Study of Drug Development. Rising protocol complexity is hindering study performance, cost, and efficiency. Impact Report: Analysis & Insight into Critical Drug Development Issues. Tufts Center for the Study of Drug Development; 2018.

[12] Center for Drug Evaluation and Research. Drug trials snapshots. U.S. Food and Drug Administration; 2019. https://www.fda.gov/drugs/drug-approvals-and-databases/drug-trials-snapshots. Accessed 7 Dec 2020.

[13] Oh S, Galanter J, Thakur N, Pino-Yanes M, Barcelo N, White M (2015). Diversity in clinical and biomedical research: a promise yet to be fulfilled. PLoS Med.

[14] Clinical Trials Transformation Initiative. CTTI recommendations: decentralized clinical trials. CTTI; 2018 (cited 21 Oct 2020). https://www.ctti-clinicaltrials.org/sites/www.ctti-clinicaltrials.org/files/dct_recommendations_final.pdf. Accessed 7 Dec 2020.

[15] van Dorn A (2020). COVID-19 and readjusting clinical trials. Lancet.

